# Stem Cell Therapy's Efficiency in Reconstructing Alveolar Clefts: A System Review and Meta-Analysis of Randomized Controlled Trials

**DOI:** 10.1155/sci/2780065

**Published:** 2025-03-30

**Authors:** Ting Li, Yang Yang Wang, Shan Li, Yunzhe Hu, Zixuan Sun, Cheng Liu

**Affiliations:** Department of Plastic Surgery, Jiangxi Provincial People's Hospital, The First Affiliated Hospital of Nanchang Medical College, Nanchang 330006, Jiangxi Province, China

**Keywords:** alveolar bone grafting, cell- and tissue-based therapy, cleft lip, cleft palate, tissue engineering

## Abstract

**Objectives:** The goal of this study was to examine the existing evidence from randomized controlled trials (RCTs) on the efficacy of cell treatment in alveolar cleft (AC).

**Design:** An electronic search was done for studies published between January 2000 and May 2024 in the PubMed/MEDLINE, Embase, Cochrane Central Register of Controlled Trials, and ClinicalTrials.gov databases. Primary outcomes were the radiographic assessment of bone graft volume, and the secondary outcome of interest was the number of complications after surgery. A random-effects model and fix-effect model were employed to pool effect sizes, and heterogeneity was assessed using *I*^2^ statistics.

**Results:** Four RCTs, comprising 51 patients, were included in the systematic review and meta-analysis. No statistically significant difference in bone volume (MD [mean difference] −0.82; 95% CI [−3.59, 5.24]; *p*=0.71) when using cells therapy to repair AC compared to using autologous iliac crest bone graft repair AC. Also, there is no difference in postoperative complications (MD 0.66; 95% CI [0.13, 3.39]; *p*=0.62) between the two groups. In this meta-analysis, cells therapy on alveolar bone grafting produced results comparable to autologous bone grafting in new bone formation rate and complications.

**Conclusions:** In conclusion, this systematic review and meta-analysis appear to indicate no disadvantage to utilizing cell therapy in AC reconstruction versus autologous bone grafting in terms of bone volume or complications.

## 1. Introduction

Since the time of Veau, efforts have been made to classify and treat clefts of the lip, palate, and alveolus, which are the most frequent congenital anomaly to affect the orofacial region [1]. Clefts in the maxillary alveolar process can interfere with facial development and symmetry as well as the eruption and maintenance of the permanent dentition [2]. The canine teeth can move and erupt through the cancellous bone thanks to alveolar bone grafting during the mixed dentition phase. According to numerous reports, autogenous bone graft taken from the ilium is thought to be the benchmark by which all other kinds of alveolar grafts should be measured [3–5]. Bone grafting success rates have typically been reported at 90%–95% [6]. However, the donor-site morbidity can reach significant levels of 8%, including significant pain, nerve damage to the lateral femoral cutaneous nerve, bleeding, and infection [4]. As alternatives to autologous bone grafts, allograft and synthetic materials can have a number of adverse effects, including decreased rates of bone growth, immune response, and infection. The need for simultaneous repair (e.g., cleft palate (CP) and alveolar cleft (AC) repair at the same time) in places where health facilities are scarce may make all of these conventional techniques more complicated.

Recently, there has been a shift toward cell-based tissue engineering approaches for alveolar bone grafting, which use cells with bone regeneration ability combined with a scaffold, such as polylactic acid, collagen, fibrin, tricalcium phosphate, and calcium carbonate [7, 8]. The effectiveness of combining cultured bone regenerative cells and biomaterials in bone defect repair has been shown in numerous animal model [9–11]. Regenerative cell therapy is a promising alternative to autologous bone grafting. However, the preparation of cultured grafts was a complicated procedure with high manufacturing costs and a risk of contamination, making it difficult to obtain regulatory approval for clinical use [12]. Autogenous osteoblast-like cells and bone marrow mononuclear cells were used for alveolar bone grafting to cut costs and preoperative time. Despite the fact that a variety of cell types were utilized in clinics, some problems remain to be resolved [13].

Several systematic reviews and meta-analyses examined the role of stem cells in alveolar bone grafting therapy. Zuk [13] first review the engineering process for craniofacial deformities may benefit from the use of adult stem cells, such as mesenchymal stem cells (MSCs) and adipose-derived stem cells. Janssen et al. [12] conducted a systematic assessment of the clinical evidence regarding the augmentation or replacement of autologous bone grafts in the AC using tissue-engineered alternatives. Furthermore, Shanbhag et al. [11] carried out a thorough systematic review and meta-analysis of cell-based tissue engineering in preclinical and clinical research across the board in all oral and maxillofacial domains. Alfayez and Alghamdi [9] conducted a systematic review of randomized clinical trials to investigate the use of stem cells for AC repair and summarize the outcomes of clinical research studies. Recently, Kalla et al. [10] conducted a system review and meta-analysis to analyze the efficacy of stem cell-based tissue engineering for the treatment of AC and CP defects in animal models. Nonetheless, none of these evaluations focused on conducting a meta-analysis limited to the reconstruction of ACs using randomized controlled trials (RCTs) with humans.

The use of regenerative cell therapy for alveolar bone grafting has resulted in poor outcomes and ongoing debate. The objective of this investigation was to evaluate the available data supporting this process, pinpoint optimal methodologies, and pinpoint other domains for superior primary research.

## 2. Methods

This systematic review followed the PRISMA (preferred reporting items for systematic reviews and meta-analyses) guidelines checklist. The protocol was registered at PROSPERO, the international prospective register of systematic reviews (Centre for Reviews and Dissemination, University of York, UK) under the number 42018085858.

### 2.1. Literature Retrieval Strategies

The meta-analysis was conducted according to the PRISMA guideline [14]. According to the principle of PICOS, the search strategies of this study were extracted: P, alveolar cleft; I, mesenchymal stem cells (MSCs) by in vitro culturing of MSCs from buccal fat pads, dental pulps, or the iliac crest; C, autologous bone graft; O: bone volume and complications; S, clinical random control trials. According to PICOS principle, the search terms are alveolar cleft, cell therapy, and iliac cancellous bone. By searching PubMed, The Cochrane Library, Embase, and the ClinicalTrials Database, the relevant literature published between January 2000 and May 2024 was searched.

### 2.2. Eligibility Criteria

Sampling of the clinical evidence was focused on RCT which related to the application of cell therapy on AC bone grafting.

The inclusion criteria are as follows:

1. English language studies.
2. Randomized controlled studies with two or more experimental groups.
3. Patients were diagnosed with the diagnosis of cleft lip and palate.
4. Transplantation of differentiated or undifferentiated MSCs seeded on biomaterial scaffolds in at least one experimental group.
5. A control group receiving autogenous bone.
6. Reported primary outcomes quantitative histomorphometric new bone formation/growth (%NBF/NBG), quantitative radiographic assessment of bone formation (BF) via computerized tomography (CT) or micro-CT (%NBF/NBG), quantitative histomorphometric assessment of remaining defect (RD), and/or quantitative radiographic assessment of RD or bone mineral density (BMD) using CT or micro-CT.
7. Reported secondary outcome is the number of complications after surgery.

The exclusion criteria are as follows:

1. In vivo or vitro studies.
2. Case reports or review studies.
3. Absence of a control group.
4. Patients were diagnosed with edentulous maxilla, atypical or nondescribed cleft diagnosis, and associated syndrome conditions.

### 2.3. Study Selection

The search results were scanned for titles, abstracts, and keywords by the two reviewers (Ting Li and Yangyang Wang), who independently and at random evaluated the titles and abstracts of all studies that appeared. All studies that seemed relevant or for which the title and abstract contained insufficient information to make a clear decision were given full copies. Two reviewers independently evaluated the full-text versions after that, and any discrepancies regarding the inclusion of studies were settled by consensus. Studies that did not fit the requirements for inclusion in this second round of selection were eliminated (Table 1).

### 2.4. Data Collection Process

Data were independently gathered by the two reviewers from the studies that were included. Information about the study's setting, the study samples' characteristics, the sources of the grafts, and the results were all extracted. The funding sources for any included studies were noted, if disclosed.  Table 2 displays the extracted information from the included studies.

### 2.5. Risk of Bias in Individual Studies

**Figure 1:**
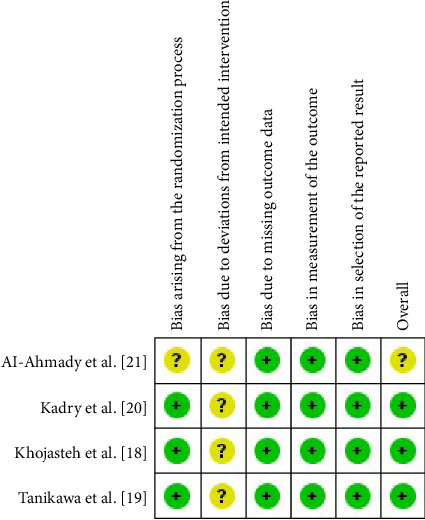
Assessment of risk of bias in the included studies.

The risk of bias in the included studies was independently evaluated by two reviewers. The tool for evaluating bias risk from the Cochrane Collaboration was utilized (Figure 1) [23]. Discussion was used to settle disagreements regarding the classification. The following domains were classified as having a “low,” “high,” or “unclear” risk of bias: bias resulting from the randomization process; bias resulting from deviations from intended intervention; bias resulting from the absence of outcome data; bias resulting from the selection of the reported result; and bias in general.

### 2.6. Summary Measures

For descriptive continuous data, mean, standard deviation (SD), sample size, and weighted mean differences (MDs) are reported. A *p* value of less than 0.05 was considered statistically significant. Random effects meta-analysis was performed using Review Manager 5.3 (Cochrane IMS, Copenhagen, Denmark) software [24]. Statistical heterogeneity was calculated by inconsistency indexes (*I*^2^), as recommended by the Cochrane Collaboration. We considered heterogeneity low if *I*^2^ was <25%, moderate if 25%–50%, and substantial if >50%.

### 2.7. Outcomes

Primary outcomes were the radiographic assessment of bone graft volume. Considering alveolar bone grafts are impacted by a variety of circumstances, and the initial conditions for inclusion studies varied, the BF ratio was finally used to assess bone quality and quantity. The BF% ratio was calculated as follows: (the postoperative formed bone volume [FV]/the volume of the actual bone graft [AV]) 100% = BF% [25]. The secondary outcome was the number of complications after surgery, including oronasal fistula, graft rejection, bone exposure, and secondary healing. The effects of different types of regenerative cells on alveolar bone grafting were compared independently. Participants' characteristics, such as average age and size of bone insufficiency, were also recorded.

### 2.8. Certainty Assessment

The quantitative synthesis and meta-included analysis studies were graded on the basis of the strength of their recommendations and the quality of the available evidence using the GRADE instrument [26]. Based on factors like study design, consistency, directness, precision, publication bias, and other elements reported by studies included in this systematic review, this assessment was made. When evaluating the evidence, tools from the website http://gradepro.org were used. The quality of the evidence was classified as high, moderate, low, or very low. Due to the small number of research (less than 10), publication bias was not evaluated using funnel plot symmetry, making the assessment techniques less trustworthy.

## 3. Results

### 3.1. Study Selection

**Figure 2:**
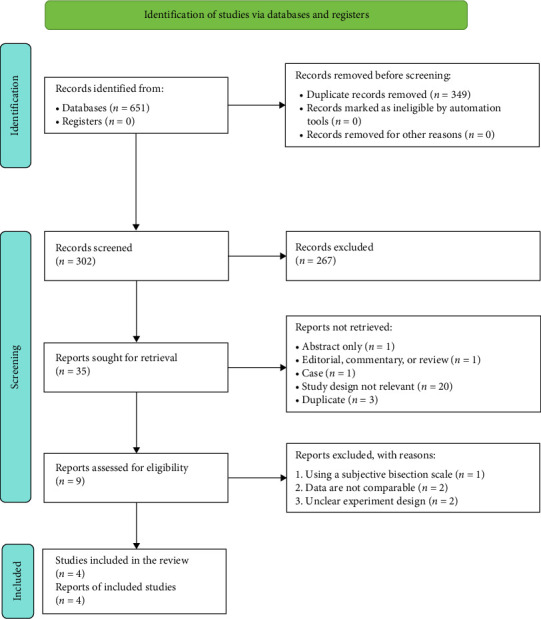
Flowdiagram.

After the search strategies, 651 publications were identified, of which 342 were excluded after reviewing the titles and abstracts. Of the remaining nine publications, full texts were obtained. After screening full texts, five studies [8, 12, 15, 16, 18] were excluded. Therefore, only four [19–22] controlled trials fulfilled all the inclusion criteria. For details of the studies examined and reasons for inclusion and exclusion, please see Tables 1 and 2. The process of study identification is presented in Figure 2.

### 3.2. Study Characteristics

A total of 51 patients with unilateral cleft lip and palate were involved, 25 underwent regenerative cell therapy for alveolar bone grafting, while 26 patients underwent iliac crest bone graft. Only 21 patients were involved in the bone volume rate comparison. Four primary articles published between 2000 and 2024 were found. All studies were randomized clinical trials. The age of patients varied between 8 and 15 years, with an average of 10 years in most studies. A primary article included unilateral and bilateral ACs; still, grafts for bilateral patients were carried out in two surgical sessions. In all articles, regenerative cell therapy for ACs reconstruction was compared to autologous bone grafting. Follow-up was carried out with a minimum of 3 months and a maximum of 12 months. Despite the fact that numerous studies indicate that the bone graft's cortical structure fully matures in 12 months and that its capacity to induce bone deteriorates after 6 months. However, because of the studies' short average follow-up, the study only evaluated the bone volume at the 6-month follow-up.

One [22] of the studies was rated as having an unclear overall risk of bias because the trial was at “unclear” risk of bias in two domains. With no more than two domains showing an unclear risk of bias, the other three studies were rated as having a low overall risk of bias. Details of the risk of bias assessment are shown in Figure 1.

### 3.3. Bone Graft Volume

**Figure 3:**
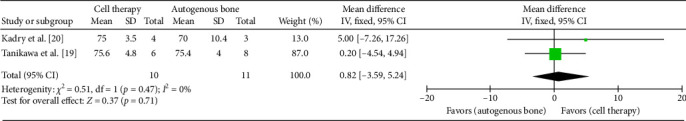
Forest plot of the meta-analysis of mean difference (MD) estimate for bone formation ratio in alveolar cleft reconstruction that regenerative cell therapy involved as the control intervention (BF%). The bone formation ratio (BF%) was calculated as follows: (the postoperative formed bone volume [FV]/the volume of the actual bone graft [AV]) 100% = BF%.

Khojasteh et al. [19] and Tanikawa et al. [20] assessed bone graft volume with radio evaluation after 6 months. Random-effect model was used to analyze the pooled data. The bone volume with regenerative cells therapy was not statistically significant higher than that with autologous iliac crest bone graft after a 6 months follow-up (MD −0.82; 95% CI [−3.59, 5.24]; *p*=0.71) (Figure 3). *χ*^2^ test for total heterogeneity was (*p*=0.47) and the *I*^2^ = 0%. Finally, the analysis of sensitivity is carried out to check the rationality of the system. The results did not change with single study exclusion.

### 3.4. Complications After Surgery

**Figure 4:**
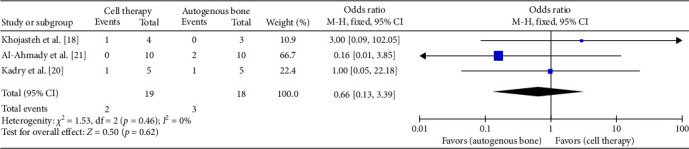
Forest plot of complications after surgery.

All of the included studies reported the complications after surgery, but only Al-Ahmady et al. [22], Kadry et al. [21], and Khojasteh et al. [19] reported the patient who had an eventful course postoperatively. Fix-effect model was used to analyze the pooled data. The analysis of the included studies did not evidence difference when using the regenerative cells therapy for the outcome complications (MD 0.66; 95% CI [0.13, 3.39]; *p*=0.62). *χ*^2^ test for heterogeneity was (*p*=0.46) and the *I*^2^ = 0% (Figure 4).

### 3.5. Certainty of the Evidence

Overall, the quality of the evidence from the outcomes evaluated by the GRADE system was assessed as low, suggesting moderate confidence in the estimated effect from the assessed outcomes. An “unclear” risk of bias among the included studies was the main factor responsible for the limited quality of the evidence (Figure 1). Publication bias was not assessed due to the limited number of studies included. An evaluation of publication bias using statistical tests or funnel plots was not carried out since each meta-analysis included fewer than 10 studies, which meant that there was insufficient power to discern genuine asymmetry from chance.

## 4. Discussion

### 4.1. Summary of Evidence

This systematic review included four clinical trials that focused on the effectiveness of regenerative cell therapy in children with cleft lip and palate after a follow-up, including clinical and radiological parameters. The main outcome was the comparison of BF ratio between regenerative cell therapy and autologous bone alone graft after a 6-month follow-up. After a 6-month follow-up, the BF rate showed no statistically significant results when applied regenerative cell therapy for alveolar bone grafting. It was suggested that regenerative cell therapy would have a comparable impact as autologous bone grafting in terms of accelerating bone regeneration. In terms of postoperative complications, the donor-site complication was not reported. These studies discovered complications such as an ornal fistula, bone exposure, partially repaired, and dehiscence over the palatally located canine crown. After a long month of follow-up, patients treated with regenerative cell therapy experienced comparable complications after surgery to patients treated with autologous bone grafting alone. In this meta-analysis, cells therapy on alveolar bone grafting produced results comparable to autologous bone grafting in NBF rate and complications. Shanbhag et al. [11] also reported that bone tissue engineering may result in comparable alveolar bone regeneration as induced by autograft.

Although only a relatively small number of studies could be included, it still enabled us to perform the meta-analyses and explore the effect of several subgroup variables. Despite this, there are some potential limitations related to this approach. The variation in the type of stem cell could be the one reason. Three types of stem cell were used in the included studies. There is still controversy on which cell source has better osteogenic potential. Mohamed-Ahmed et al. [27] in rat skull defects showed that despite the potential of ASC to regenerate bone, ASC may regenerate bone slower than BMSC, and BMSC is more suitable for bone regeneration applications. Laboratory studies by Huang et al. [28] also showed that ADSCs in mice showed similar osteogenic differentiation capacity to BMSCs, but had better ability than BMSCs in terms of stem cell purity and cell proliferation.

Also, different type of scaffolds was shown in the included studies. Three categories are commonly used to categorize scaffolds: ceramics, synthetic polymers, and natural polymers [29]. Ceramic scaffolds are among the materials most frequently utilized in bone regeneration because of their strong osteoconductivity and biocompatibility. By altering the composition, it is possible to regulate even the rate of their disintegration. Synthetic polymers, however, emit acidic chemicals during decomposition, which may have a detrimental effect on the healing tissue. Natural polymer scaffolds indicate that the primary components are sourced from natural sources and have the potential to stimulate stem cells through the delivery of growth factors. However, natural polymers lack sufficient mechanical characteristics yet do not generate harmful byproducts and are biocompatible. To increase their mechanical properties, they can be transformed into biomimetic scaffolds by incorporating, for instance, ceramic phase. The purpose of producing synthetic polymers was to provide them with good mechanical qualities and the capacity to stimulate stem cells. However, neither of the advancements is equivalent to natural polymers or ceramics polymers.

Defect size also influences the clinical application of cell-based tissue engineering [30–32]. Unlike calvarial critical-size defects, alveolar critical-size defect models have not been well characterized in the literature regarding defect location, size, and morphology. Defect dimensions varied between studies for the same animal model/species. As was previously said, the timing of the procedure is quite important. Regardless of the degree of the cleft, the success rate is higher and comparable when an alveoloplasty is done prior to canine eruption. The severity of the cleft has an adverse effect on surgical success, and late alveoloplasty (after canine eruption) has a lower success rate. Studies have shown that clefts greater than 10 mm and bilateral clefts may increase the risk of problems or reaching type 3–4 on the Bergland scale by 4–6 times as compared to when they are absent [31].

According to the GRADE working group, the quality of the evidence in this research was graded as moderate, which means the conclusions of this review should be evaluated with caution and more RCTs would be needed for further evaluation. Because of the substantial bias in the current study and the limited number of articles, more high-quality RCT trials are needed to examine the efficacy of PRC in alveolar bone grafting.

## 5. Conclusion

In conclusion, this systematic review and meta-analysis appear to indicate no benefit to utilizing cell therapy in AC reconstruction versus autologous bone grafting in terms of bone volume or complications.

## Figures and Tables

**Table 1:** Characteristics of the excluded studies.

Study	Reason for exclusion
Behnia et al. [15]	It is not a random control trial and just three patients in it
Bajestan et al. [8]	No information about the initial bone volume and bone volume
Mossaad et al. [16]	Only given the bone density at 6 months follow-up with gm/cm^2^
Du et al. [17]	It did not randomly allocate patients to cell therapy and autogenous bone graft treatments
Pradel and Lauer [18]	It did not randomly allocate patients to cell therapy and autogenous bone graft treatments

**Table 2:** Characteristics of included studies.

Author (year)	Study design, type of patient (duration)	Type of stem cell	Type of scaffold	Outcome assess measure	Age (mean/range)	Study group (*n*), defect volume (cm^3^)	Complications
Khojasteh et al. (2017) [19]	RCT, UCLP (6 months)	Buccal fat pad derived stem cells	Natural bovine bone mineral	CT	8–14	BMMSCs/LRCP (*n* = 4) Size: 0.008 ± 0.003 ICBG (*n* = 3) Size: 0.007 ± 0.002	One patients bone exposure in TF

Tanikawa et al. (2020) [20]	RCT, UCLP (12 months)	Deciduous dental pulp stem cells	Bio-Oss Collagen	CT	8–12	DDPSC/Bio-Oss Collagen (*n* = 6) Size: 1.0286 ± 0.213 ICBG (*n* = 8) Size: 1.0524 ± 0.326	None

Kadry et al. (2021) [21]	RCT, UCLP (12 months)	Bone marrow−derived mesenchymal stem cells	Collagen	CT	9–11	BM-MSCs/collagen (*n* = 5) Size: not mention ICBG (*n* = 5) Size: not mention	One patients bone exposure in ICBG. One patients dehiscence over the palatally situated canine crown in TF

Al-Ahmady et al. (2018) [22]	RCT, UCLP (12 months)	Bone marrow mononuclear cells	Collagen sponge	CT	8–15	BMMNCs/*β*-TCP + PRF (*n* = 10) Size: not mention ICBG (*n* = 10) Size: not mention	Two patients ornal fistula in ICBG

Abbreviations: CT, computerized tomography; RCT, randomized controlled trial.

## Data Availability

Data sharing is not applicable to this article as no datasets were generated or analyzed during the current study.
